# Alcohol’s Effects on the Adolescent Brain: What Can Be Learned From Animal Models

**Published:** 2004

**Authors:** Susanne Hiller-Sturmhöfel, H. Scott Swartzwelder

**Affiliations:** Susanne Hiller-Sturmhöfel, Ph.D., is senior science editor for *Alcohol Research & Health*. H. Scott Swartzwelder, Ph.D., is a professor in the Department of Psychiatry and Behavioral Sciences at Duke University Medical Center and a senior research career scientist at the Durham Veterans Affairs Medical Center, both positions in Durham, North Carolina

**Keywords:** adolescent, binge drinking, animal study, animal model, physiological AODE (alcohol and other drug effects), biological development, brain, brain function, cognition, learning, memory, motor coordination, AODR (alcohol and other drug related) seizure, hippocampus, neurotransmitter receptors

## Abstract

Because of legal and ethical constraints on alcohol research in human adolescents, many studies of alcohol’s effects on the developing brain have been conducted in animal models, primarily rats and mice. The adolescent brain may be uniquely sensitive to alcohol’s effects because major changes in brain structure and function occur during this developmental period. For example, adolescent animals are more sensitive than adults to the effects on memory and learning that result from alcohol’s actions on the hippocampus. Conversely, adolescent animals appear to be less sensitive than adults to alcohol-related motor impairment, alcohol-induced sedation, and the development of seizures during withdrawal. Alcohol exposure during adolescence can have long-lasting effects and may interfere with normal brain functioning during adulthood.

Adolescence and young adulthood are developmental stages of transition during which humans, as well as members of many other species, mature physically and behaviorally into their adult state. Adolescents and young adults need to acquire the physical and behavioral skills that will allow them to live independently of their parents, sustain themselves, and reproduce. This period is marked by more frequent and sophisticated social interactions with peers, exploration of new situations and behaviors, and an increased willingness to take risks. In humans, this often involves the initiation of alcohol and other drug use.

At the same time, the brain undergoes considerable structural and functional changes, at least in part in response to the individual’s many new experiences. Connections among nerve cells (neurons) in the brain can change based on which neurons or groups of neurons are regularly stimulated, a characteristic known as plasticity. This natural process serves to eliminate unnecessary or unused nerve cell connections,^1^ allowing the survival of only those neurons that make meaningful contacts with other neurons. This winnowing of neurons is influenced by, among other factors, the adolescent’s interactions with and experiences in the outside world.

Adolescence is such a critical phase in brain development that the actions of alcohol and other drugs on the brain can be assumed to have a particularly profound impact during this developmental period. Indeed, research has shown that compared with the adult brain, the adolescent brain is particularly sensitive to some effects of alcohol, yet more resistant to other effects. Much of this research, especially investigations of specific effects of acute alcohol administration, has been conducted in animals because studies involving administration of alcohol to human adolescents are subject to very stringent regulations, and certain studies of alcohol’s effects on the adolescent brain can be conducted only using animal models. This article reviews some of the differences in alcohol’s effects on the adolescent and adult brain that were identified using these animal models. The accompanying article by Tapert and colleagues (pp. 205–212) summarizes information that has been obtained in studies of human adolescents and young adults.

## Major Changes in Brain Structure and Function During Adolescence

Adolescence in humans is broadly defined as the second decade of life, although some researchers consider ages up to 25 years as “late adolescence.” The corresponding period in laboratory animals that are frequently used as study subjects is just as loosely defined. In rats it typically spans postnatal days 30–50 (i.e., PD30–PD50). In both humans and animal models, adolescence is a period when the brain undergoes extensive remodeling. New connections among neurons are being formed; at the same time, a substantial number of existing connections are lost (see Spear 2000). It is hypothesized that this plasticity allows the individual’s brain to be sculpted based on his or her personal experiences and interactions with the outside world (Chugani 1998).

One brain region where particularly extensive remodeling occurs is the frontal region of the outer layer of the brain—the prefrontal cortex—which is thought to be involved in working memory, voluntary motor behavior, impulse control, rule learning, spatial learning, planning, and decisionmaking (see Spear 2000; White and Swartzwelder 2005). These changes are especially extensive in humans. Although the number of neurons and neuronal connections in the prefrontal cortex appear to decline during adolescence, the relative importance of the frontal lobes increases.

Developmental changes in the behavioral relevance of certain brain areas are accompanied by increases or decreases in the activities of chemicals called neurotransmitters, which help transmit nerve signals from one neuron to another. This signaling takes place when neurotransmitters released by one neuron bind to protein molecules called receptors on the surface of the receiving neuron. The interaction between the neurotransmitter and its receptor initiates chemical and electrical changes in the signal-receiving neuron that influence the generation of a new nerve signal in that cell. In this way, nerve cells and circuits communicate and drive behavior. Excitatory neurotransmitters promote the generation of new nerve signals, whereas inhibitory neurotransmitters make it more difficult to generate a nerve signal in a signal-receiving neuron. Numerous neurotransmitters and their receptors have been identified that act on specific cells or groups of cells and have specific effects on those cells.

Two important neurotransmitter systems that undergo substantial changes during adolescence and are affected by alcohol consumption are dopamine and gamma-aminobutyric acid (GABA). Dopamine can have both excitatory and inhibitory effects, depending on the cells it acts on. Dopamine-releasing and dopamine-receiving cells are found in numerous brain areas. One prominent region, which lies deep within the brain, is called the striatum. It consists of several components that are involved in behaviors such as learning to automatically execute complex movements triggered by a voluntary command (e.g., driving a car). Another dopamine-using area is the nucleus accumbens, which plays a role in learning and performing certain behaviors in response to incentive stimuli (i.e., motivation) (Di Chiara 1997). Activity in the nucleus accumbens in part accounts for the fact that people perceive the effects of drinking alcohol or taking other drugs as pleasurable (Di Chiara 1997).

During adolescence, the dopamine system in the striatum appears to undergo substantial changes. For example, studies in rats have found that dopamine receptor levels in the striatum increase during early adolescence but then decrease during late adolescence and young adulthood (Teicher et al. 1995). At the same time, dopamine receptor levels in the nucleus accumbens increase dramatically.

GABA is the primary inhibitory neurotransmitter in the brain—that is, it represses the activity of other brain cells. Alcohol generally enhances the effects of GABA on its receptors. This enhanced GABA activation may play a role in mediating the sedative effects of alcohol and other sedating agents (Mihic and Harris 1997). In addition, alcohol’s effects on GABA and its receptors are thought to contribute to the development of tolerance to and dependence on alcohol (Mihic and Harris 1997). Like dopamine, the GABA system changes substantially during adolescence. Studies in rats have found that the number of GABA receptors, and thus the activity of the GABA system, increases markedly in several brain structures during early adolescence (Moy et al. 1998).

In addition to these two neurotransmitter systems, a system using the neurotransmitter glutamate also appears to undergo changes during adolescence. Glutamate interacts with several receptors, including one called the NMDA receptor. Evidence from animal studies indicates that the NMDA receptor complex changes during postnatal development, and these changes may continue into adolescence (McDonald et al. 1990).

Although it is beyond the scope of this article to review the changes occurring in various brain structures and neurotransmitter systems in more detail, this brief description demonstrates that adolescence is a period of profound alterations in brain function. Therefore, it is reasonable to expect that alcohol’s effects on the brain and behavior may differ for adolescents and adults. The following sections will review some of the differences in sensitivity to alcohol that have been identified using animal models.

## Adolescents Are More Sensitive Than Adults to Alcohol’s Memory-Impairing Effects

### Alcohol’s Effects on Memory

Among its many effects on the brain and brain function—such as impairing balance, motor coordination, and decisionmaking—alcohol interferes with the drinker’s ability to form memories (i.e., it is an amnestic agent). However, alcohol does not impair all types of memory equally. Alcohol disrupts a person’s ability to form new, lasting memories to a far greater extent than it interferes with the ability to recall previously established memories or to hold new information in memory for just a few seconds (see White and Swartzwelder 2004). One study conducted with young adults ages 21 to 29 found that intoxicated study participants could recall items on word lists immediately after the lists were presented, but they had greater difficulty recalling the information 20 minutes later (Acheson et al. 1998). Interestingly, this effect was much more powerful in the younger subjects in this age group—that is, people in their early twenties. In addition, alcohol particularly affects the ability to form explicit memories—that is, memories of facts (e.g., names and phone numbers) or events (e.g., what the drinker did the previous night). Because different brain areas play a role in the formation of different types of memories, this pattern of alcohol-related memory impairment allows researchers to make assumptions about the brain regions that are most affected by alcohol. Thus, the pattern of memory impairment observed after intoxication is similar to that found in patients with damage to a brain area called the hippocampus.

### Alcohol and the Hippocampus

**Figure 1:**
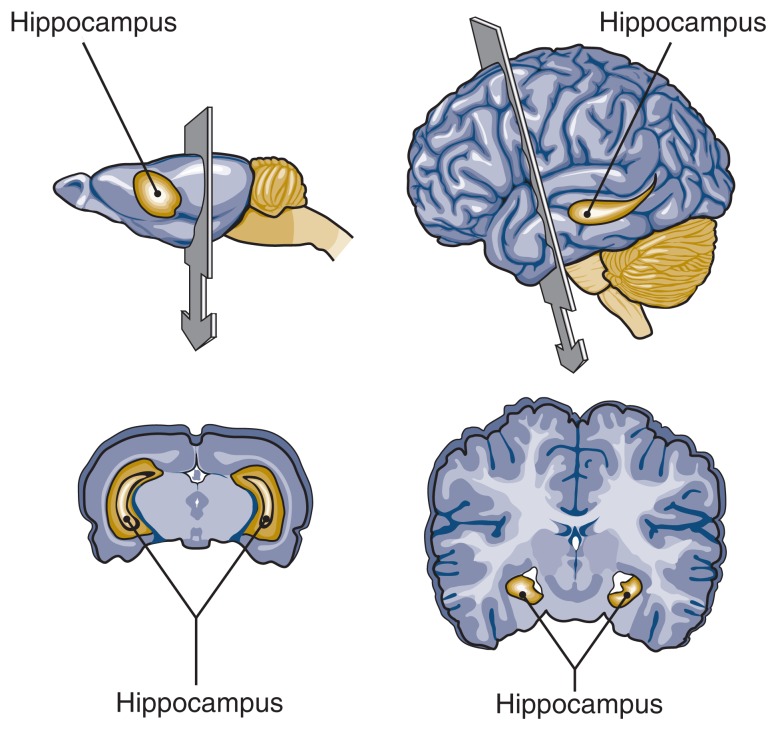
Location of the hippocampus, an area of the brain that appears to be particularly vulnerable to alcohol’s effects. It sits below the surface of the neocortex in the rat brain (left) and the human brain.

The hippocampus is located deep under the brain’s surface (see figure 1) but is extensively connected with the outer layer of the brain (i.e., the neocortex). It consists of only a few layers of cells arranged in a characteristic shape with several bends and folds. The primary cells in the hippocampus are called pyramidal cells because of their shape. The hippocampus can be divided into several areas, and studies in humans have found that in some patients with an inability to form new explicit memories, brain damage was limited to a single region of hippocampal neurons called the CA1 region (Zola-Morgan et al. 1986). In rodents, the activity of CA1 cells correlates strikingly with behavior: Each CA1 neuron tends to emit signals primarily when the animal is in a specific area of its environment. For example, cell A may be active predominantly when the animal is in the northeast corner of its cage, whereas cell B may become active when the animal enters the southwest corner of the cage. As a result, these cells can play a very strong and specific role in spatial learning (e.g., the ability to learn the path through a maze or the location of a certain item, such as a food reward).

Researchers have used this characteristic of the CA1 cells to assess the effects of alcohol exposure and other interventions on hippocampal cell activity in intact, living rodents. In one study, electrodes were implanted in the hippocampus of rats that then were able to move freely around their cages. After the animals were administered alcohol, the activities of their CA1 cells were measured. This study found that the activity of the CA1 cells was reduced when alcohol levels reached at least 0.5 grams per kilogram (g/kg) of body weight and ceased almost completely at higher alcohol doses (White and Best 2000). This finding is consistent with the hypothesis that alcohol can interfere with the formation of new explicit memories by disrupting hippocampal function.

### Alcohol’s Effects on Long-Term Potentiation

**Figure 2:**
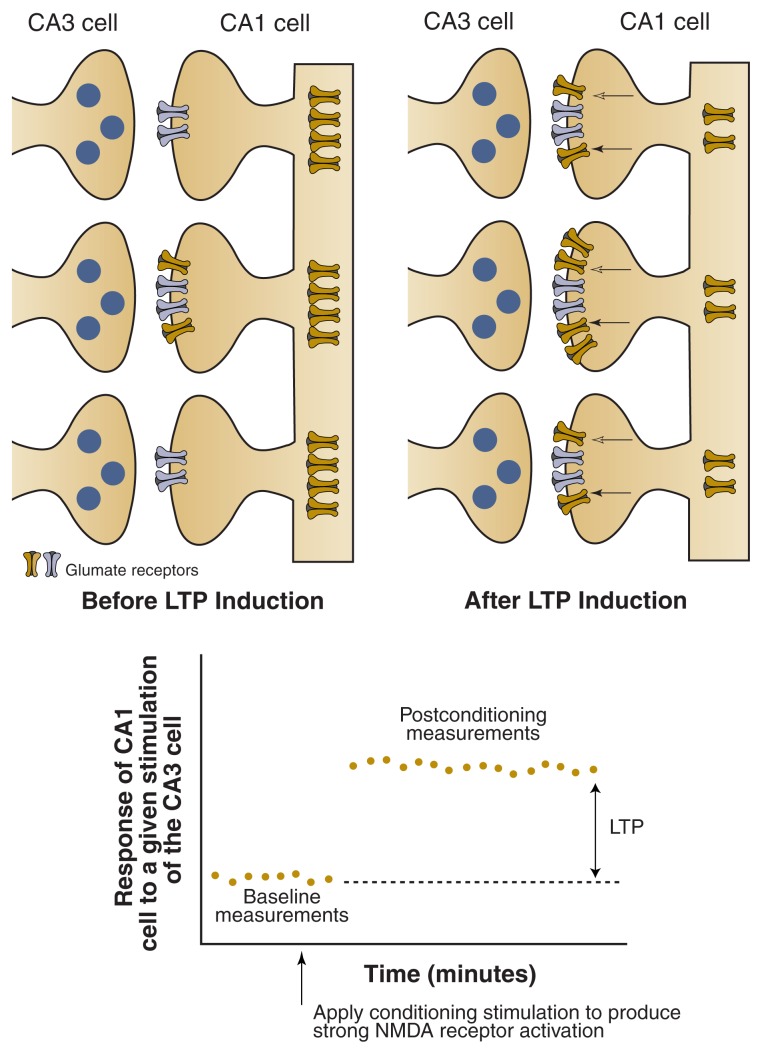
Schematic representation of the long-term potentiation (LTP) process. When a hippocampal CA3 cell is initially stimulated, it releases the neurotransmitter glutamate, which binds to NMDA receptors on a CA1 cell and induces a response of a certain size (baseline response). One mechanism underlying the induction of LTP may be that when the CA3 cell is repeatedly stimulated in the proper pattern, the number of glutamate receptors on the CA1 cell increases and the receptors become activated. If the original stimulus is then reapplied to the CA3 cell, the resulting glutamate release will induce a much greater response in the CA1 cell. This is called long-term potentiation.

In addition to interfering with the activity of CA1 cells, alcohol can impair other hippocampal functions. One of these, a process called long-term potentiation (LTP), is an experimentally induced adaptation of the nerve cell connections in response to repeated activation or stimulation of these connections.^2^ To illustrate, imagine two neurons in the hippocampus—a CA1 neuron and a neuron from a region called CA3—that connect in the hippocampus, with the CA3 neuron sending signals to the CA1 neuron. To transmit the signals, the CA3 neuron releases a neurotransmitter, which then interacts with receptors on the surface of the CA1 neuron,^3^ resulting in the formation of a new nerve signal in the CA1 neuron (see figure 2). The intensity of this signal depends on various factors, including the number of receptors on the CA1 neuron. When the CA3 neuron first is exposed to a stimulus, it will emit a signal that leads to a certain level of response in the CA1 neuron. This is called the baseline response. The CA3 neuron then can be stimulated experimentally in a specific pattern, a process that resembles what happens during actual learning events. If the original stimulus subsequently is reapplied to the CA3 neuron, it will evoke a response in the CA1 neuron that is substantially greater than the response that occurred after the initial stimulation (i.e., the response is potentiated). In other words, as the result of the patterned stimulation, the CA1 cell becomes more responsive to signals emitted by the CA3 cell. This potentiated response often persists for a long period of time, hence the name “long-term” potentiation. There is accumulating evidence that something like LTP occurs naturally during learning and memory formation.

Alcohol has been shown to interfere with LTP during experiments using hippocampal brain slices from rats. In these experiments, alcohol concentrations corresponding to those achieved in humans after consuming only one or two drinks interfered with the establishment of LTP (Blitzer et al. 1990). The brain slices were kept in a special fluid, and two electrodes were introduced into the tissue, one that allowed stimulation of the CA3 cells and one that recorded the responses of the CA1 cells. If sufficient alcohol was present in the surrounding fluid during the repeated patterned stimulation of the CA3 cells, LTP was not detected in the CA1 cells—that is, their response remained at the baseline level. However, adding alcohol to the fluid after the patterned stimulation had no effect on LTP, which is consistent with the observation that alcohol consumption does not impair recall of previously established memories. Although experiments like this make it tempting to equate LTP with actual learning, it is important to remember that LTP really is a manifestation of neural plasticity that shares some common mechanisms with learning. Even though actual learning is certainly more complex than simple LTP induced in the lab, the LTP process represents an excellent opportunity to study the brain mechanisms underlying memory and the effects of drugs such as alcohol on these mechanisms.

One neurotransmitter system involved in the establishment of LTP is the excitatory neurotransmitter glutamate and its NMDA receptor. When this receptor is activated by glutamate, it allows calcium to enter the cells. Repeated calcium influx, in turn, sets off a chain reaction leading to long-lasting changes in the structure and/or function of the cells that cause LTP. Alcohol has been shown to interfere with activation of the NMDA receptor, thereby reducing calcium influx and, thus, the subsequent changes in cell function that result in LTP. Researchers think that this is the main mechanism through which alcohol prevents establishment of LTP, although other neurotransmitter systems also may play a role (see White and Swartzwelder 2004).

### Differential Effects of Acute Alcohol on Memory in Adolescents and Adults

Some evidence suggests that alcohol’s effects on memory and learning are much more severe in adolescents than in adults. Although difficult to assess in humans, age differences in alcohol’s effects on memory can be studied in rodents. One approach uses a test called the Morris water maze task. In this type of experiment, animals are placed in a large circular tank filled with opaque water. The animals must then locate a platform, submerged about an inch beneath the surface, where they can rest. The ability to remember the location of the platform across repeated trials requires activity of the hippocampus; thus, changes in hippocampal function can be detected by measuring the animal’s ability to learn the location of the platform.

To assess age-dependent effects of alcohol, Markwiese and colleagues (1998) compared the performance of alcohol-exposed adolescent and adult rats in the Morris water maze task. Each animal underwent 5 days of training to learn the location of the platform. Before each training session, one group of animals received no alcohol, and two other groups received one of two different alcohol doses. The investigators then compared how long it took the alcohol-exposed and control animals to remember the location of the platform. Among the adult animals, only those exposed to the highest alcohol concentration showed learning impairments compared with the control group. In contrast, adolescent animals also showed impairments after they had received the lower alcohol dose (Markwiese et al. 1998). This experiment demonstrates that adolescent rats are more vulnerable to alcohol’s effects on memory and learning than are adult rats. It is not known if the same age-related difference exists in humans, as corresponding experiments in human adolescents cannot be done for obvious reasons. However, as mentioned previously, one study comparing people in their early twenties with people in their late twenties found that the younger age group seemed more vulnerable to alcohol–induced memory impairment (Acheson et al. 1998).

Researchers also have investigated the mechanisms underlying age-related differences in sensitivity to alcohol’s effects on memory. These analyses have demonstrated that alcohol-induced inhibition of LTP and of NMDA receptor–mediated activity were greater in brain slices from adolescent rats than in brain slices from adult rats. For example, in studies using hippocampal slices taken from adolescent and adult rats, repeated stimulation in the absence of alcohol induced LTP in samples taken from both age groups (Swartzwelder et al. 1995*a*; Pyapali et al. 1999). In fact, in the absence of alcohol, the LTP was more pronounced in adolescent than in adult brain tissue. When alcohol was added, however, LTP induction was reduced substantially or almost completely blocked in the adolescent tissue, whereas it took much higher alcohol concentrations to inhibit the LTP process in tissue from adults.

Similar experiments compared the activity of the glutamate/NMDA system in response to stimulation in the presence or absence of alcohol in hippocampal brain slices from adolescent and adult rats. It took significantly higher concentrations of alcohol to reduce NMDA receptor activity in the adult brain slices, compared with those taken from adolescent animals (Swartzwelder et al. 1995*b*).^4^

All of these studies confirm the heightened susceptibility of the adolescent rodent brain to alcohol-induced inhibition of hippocampal function and memory formation.

## Adolescents Are Less Sensitive Than Adults to Other Alcohol Effects

As the preceding section has shown, adolescent animals are uniquely sensitive to some of alcohol’s effects on memory. However, adolescents seem less sensitive than adults to other effects of drinking, such as impairment of motor coordination, sedation, and susceptibility to seizures during withdrawal.

### Motor Coordination

One of the most obvious effects of alcohol consumption in humans as well as laboratory animals is the impairment of motor activity and coordination. Alcohol interferes with a person’s ability to perform tasks that require balance and motor coordination, such as standing still, walking in a straight line, or driving an automobile. In animals, alcohol’s effects on motor coordination can be demonstrated using the tilting plane test, in which an animal is placed on a horizontal platform that is gradually tilted, so that the animal must adjust its position to maintain its balance.

Motor coordination is one of the primary functions of the cerebellum, an area at the base of the brain. Because the cerebellum continues to develop during adolescence, it is reasonable to assume that alcohol might affect motor coordination in adolescents differently than in adults. To investigate this possibility, White and colleagues (2002*a*) analyzed the motor coordination of adolescent and adult rats using the tilting plane test before, and at various time points after, administering alcohol at three different doses (1.0, 2.0, and 3.0 g/kg body weight). These researchers found that the lowest alcohol dose did not affect the animals’ performance in either age group. At almost all time points after the administration of the two higher doses, however, the adult animals were more impaired than the adolescent animals. These findings clearly demonstrate that, in contrast to alcohol’s effects on memory, adolescent rats appear to be less sensitive to alcohol’s effects on motor coordination than adult rats. It is not clear precisely why the adolescent animals were less sensitive to alcohol-induced motor impairment. It is clear, however, that the cerebellum, which plays a critical role in motor coordination, still is developing quite rapidly during adolescence. If the cerebellum is less sensitive to alcohol during this period, this could account for the developmental difference in sensitivity to alcohol. Currently, it is not known if this difference in sensitivity also applies to human adolescents.

### Sedation

Another common effect of alcohol consumption that can be observed both in humans and in animals is sedation. With increasing alcohol consumption, drinkers tend to become sleepy and eventually may even pass out. In laboratory animals, sedation can be assessed by observing the righting reflex that normally helps the animals get back on all four feet if they fall over. When treated with sedative agents, animals temporarily lose this righting reflex, and the duration of this loss is a measure of the sedative potency of an agent.

To better characterize alcohol’s effects on the developing organism, researchers also have evaluated alcohol’s sedative effects in rats of different ages. Little and colleagues (1996) injected animals from three age groups—juvenile animals (PD20), adolescent animals (PD30), and adult animals (PD60)—with three different alcohol doses, and found the following:

- When treated with the lowest alcohol dose (3 g/kg body weight), none of the adolescent animals lost their righting reflex, whereas one-half of the juvenile rats and two-thirds of the adult rats did.
- Adolescent animals lost the righting reflex for a significantly shorter period of time than adult animals. When they regained the reflex, adolescent animals also had significantly higher blood alcohol concentrations than the adult animals had when they regained the righting reflex.
- The juvenile animals also lost the righting reflex for a significantly shorter time than the adult animals, although not as short as the adolescent animals.

These observations demonstrate that, as with alcohol’s motor-impairing effects, adolescent animals are substantially less sensitive to alcohol’s sedative effects. Mechanisms that may contribute to this lower sensitivity are discussed in the following section.

### Mechanisms That May Contribute to Reduced Motor-Impairing and Sedative Effects in Adolescents

Researchers have not yet identified the mechanisms that account for the fact that adolescents are less susceptible to alcohol-related motor impairment and sedation than older individuals. It is likely, however, that the neurotransmitter GABA and its receptors play a role in both of these effects. As mentioned earlier, GABA is an inhibitory neurotransmitter, and the activity of GABA and its receptors is enhanced by alcohol. As a result, the GABA system has been implicated in both alcohol’s sedative and its motor-impairing effects. Studies using rats have found that the levels of GABA receptors in various brain structures, including the cerebellum, increase markedly throughout adolescence and reach their final levels during early adulthood (Moy et al. 1998). Thus, it appears possible that adolescent rats are less sensitive to alcohol-induced motor impairment and sedation because, compared with older animals, they have fewer GABA receptors on which alcohol can act. Another possibility is that the function of GABA receptors is altered across adolescent development in a way that results in increased sensitivity to alcohol as the animal gets older. The combination of reduced sensitivity to alcohol’s motor-impairing and sedative effects on the one hand and increased sensitivity to alcohol’s memory-impairing effects on the other hand could be particularly harmful to adolescents. For most people, the maximum amount of alcohol they can consume is determined by alcohol’s motor-impairing and sedative effects—that is, if they do not stop drinking voluntarily, drinkers at some point become so incapacitated that they cannot continue to drink even if they want to. If, like adolescent animals, human adolescents also are less sensitive to these alcohol effects, it appears plausible that they might continue to drink longer than adults, achieving higher blood alcohol concentrations in the process. As a result, the adolescents could become even more vulnerable to the effects of alcohol on memory and other functions to which they are more susceptible than adults even at lower blood alcohol levels.

### Susceptibility to Seizures During Withdrawal

Like all neurotransmitters, GABA has numerous functions and effects in regulating brain activity. For example, in addition to playing a role in motor impairment and sedation, GABA also is involved in the development of seizures during alcohol withdrawal. Long-term drinking causes the body to adjust to the continued presence of alcohol so that it eventually functions normally only in the presence of the drug. At that point, cessation of drinking can lead to an array of adverse symptoms, collectively called withdrawal, which include symptoms mediated by GABA.

Because alcohol stimulates the activity of GABA receptors, long-term drinking causes the brain to produce fewer of these receptors. If alcohol then is withheld, GABA activity suddenly drops off because fewer GABA receptors are available and alcohol no longer activates the ones that remain. This insufficient GABA activity has been linked to the development of seizures during withdrawal. If adolescents are less sensitive than adults to alcohol’s effects on GABA and its receptors, adolescents also should be less prone to seizures during withdrawal from alcohol.

This hypothesis has been investigated in rats. For these experiments, Acheson and colleagues (1999) administered alcohol to adolescent and adult rats for 5 days, then injected the animals with a chemical that induces seizures and rated the severity and duration of the seizures. The study found that although adolescent and adult animals experienced seizures of various severities at a similar rate, the more severe seizures lasted significantly longer in the adult animals than in the adolescent animals. Thus, this study supports the hypothesis that adolescent animals are less sensitive than adults to alcohol’s effects on the GABA system.

## Alcohol Exposure During Adolescence Affects Brain Function During Adulthood

When investigating alcohol’s effects on the adolescent brain, it is important not only to focus on the immediate effects (e.g., memory impairment, motor impairment, or sedation) but also to explore the consequences of alcohol use on the adolescent’s future development. Because the brain undergoes such extensive changes and remodeling during adolescence, it is reasonable to assume that disruption of these processes by alcohol could lead to long-term alterations that influence adult behavior and responses to alcohol.

The preceding sections have described how acute alcohol exposure affects the body differently during adolescence than during adulthood, with adolescents being more sensitive to some effects of alcohol and less sensitive to others. In addition, adolescents may respond differently to repeated heavy alcohol exposure, a drinking pattern also known as chronic intermittent exposure or binge drinking, which is particularly common among adolescents. Binge drinking is characterized by repeated episodes of heavy drinking followed by withdrawal. Several lines of evidence suggest that these repeated withdrawal episodes contribute to many of the effects of chronic alcohol exposure on the brain (see White and Swartzwelder 2004).

In one study of the long-term consequences of binge drinking during adolescence, White and colleagues (2002*b*) studied animals that were repeatedly exposed to high levels of alcohol during adolescence. The alcohol-exposed and control animals were evaluated as adults with respect to alcohol’s effects on motor activity, using the tilted plane test. As mentioned earlier, adult rats normally are more sensitive than adolescents to alcohol-induced motor impairment (i.e., the rats’ sensitivity to motor impairment increases between adolescence and adulthood). This study found, however, that rats repeatedly exposed to alcohol during adolescence did not show this increase in sensitivity to alcohol’s effects (White et al. 2002*b*); these animals performed as well on the tilted plane test in adulthood as they had in adolescence. In a control experiment, adult rats were exposed to the same regimen of alcohol administration as were the adolescent animals. When these adult rats were subsequently tested, their sensitivity to alcohol-induced motor impairment was unchanged despite the repeated alcohol exposure. Thus, it is not the alcohol treatment per se that leads to reduced sensitivity to motor impairment; instead, it appears that alcohol exposure during adolescence interferes with the developmental processes that lead to adult sensitivity to alcohol’s effects on motor coordination.

In a similar experiment, White and colleagues (2000) evaluated how chronic intermittent alcohol exposure during adolescence affects rats’ spatial memory in adulthood. As discussed earlier, acute alcohol administration impairs learning and memory more in adolescent animals than it does in adults. For this experiment, adolescent and adult animals were repeatedly exposed to high doses of alcohol. When all the animals had reached adulthood, the investigators compared their ability to learn where to retrieve food in a maze with that of animals which had never received alcohol. They found that animals in all test groups (i.e., with or without alcohol administration during adolescence or adulthood) learned to perform the memory task equally well. However, when the animals received a low dose of alcohol just before being tested on the memory task, those that had been exposed to alcohol as adolescents performed worse than animals in the other three groups (White et al. 2000). These results indicate that repeated alcohol exposure during adolescence enhances the individual’s sensitivity to alcohol’s memory-impairing effects during adulthood. Similar results were obtained in a study of college students, which found that students with a history of binge drinking performed worse on memory tasks after consuming alcohol than did students without such a history (Weissenborn and Duka 2003).

Researchers also have demonstrated the long-term consequences of adolescent alcohol exposure on adult brain function by measuring the electrical brain activity of adult rats that had or had not been repeatedly exposed to alcohol during adolescence. Using electrodes implanted in various regions of the animals’ brains, researchers examined both the electroencephalogram (EEG), which is a measure of ongoing brain activity, and event-related potentials (ERPs), which are spikes in brain activity induced by a sudden stimulus (e.g., a light or sound). One of the studies found that animals which had been exposed to alcohol during adolescence showed changes in the EEG pattern as well as in ERPs measured in various brain regions, particularly the hippocampus (Slawecki et al. 2001). These investigators noted that although similar effects have been reported following long-term alcohol exposure during adulthood, alcohol exposure during adolescence appears to result in more stable effects, especially on the hippocampus, after shorter periods of exposure than would be observed in adult animals.

Similar experiments have examined the effects of an acute alcohol dose on the EEG of adult rats that had or had not been exposed to alcohol repeatedly during adolescence. A study by Slawecki (2000) found that although the acute alcohol dose significantly altered several EEG variables in the hippocampus and other brain regions of the control animals, these variables were not altered in the animals that had been exposed to alcohol during adolescence. In addition, the alcohol-exposed animals showed fewer behaviors indicative of intoxication in response to the acute alcohol dose than did the control animals. These findings suggest that alcohol exposure during adolescence leads to persistent and brain region–specific changes in electrical brain activity in response to an acute alcohol dose during adulthood. In particular, the observation that some EEG responses to alcohol were reduced in the alcohol-exposed animals indicates that adolescent alcohol exposure can produce long-lasting changes in responsiveness to at least some alcohol effects.

## Conclusions

Various avenues of research have demonstrated that at least in laboratory animals, adolescence is a unique stage of brain development which is particularly sensitive to the disrupting effects of alcohol. For example, in rodents, adolescent alcohol exposure increases the brain’s sensitivity to some alcohol effects (e.g., memory impairment) and decreases sensitivity to other effects (e.g., motor impairment and sedation). Furthermore, in rodents, alcohol exposure during adolescence not only has an immediate impact on brain function, it also may lead to consequences for various brain functions that last even into adulthood. To what extent these findings are applicable to humans is a matter of debate, particularly because of the differences between humans and rodents in terms of the plasticity and time course of brain development. Nevertheless these findings suggest that similar processes might occur in humans—a conclusion that is especially pertinent and worrisome because adolescence in humans often is the period when alcohol consumption begins and when particularly dangerous drinking patterns, such as binge drinking, are common. This combination of frequent high alcohol consumption and increased vulnerability of the brain to alcohol’s harmful effects may result in cognitive deficits and other problems that persist far beyond adolescence.

One brain area that seems to be particularly affected by adolescent alcohol consumption is the hippocampus, which plays a role in numerous cognitive functions, including learning and memory. In fact, preliminary studies in humans have found that alcohol abuse during adolescence may be associated with a reduction in the size of the hippocampus (De Bellis et al. 2000), which in turn could be a sign of impaired hippocampal function. Theoretically, alcohol could lead to cell death in the hippocampus through several mechanisms (e.g., by excessive activation of the glutamate/NMDA receptor system). Several studies, however, have failed to detect obvious nerve cell loss after repeated exposure to various patterns of alcohol administration during adolescence or early adulthood (see White and Swartzwelder 2004). Other studies, in contrast, have found that high-dose binge exposure to alcohol led to brain damage in adolescents but not in adults (Crews et al. 2000). These differences in findings may be accounted for by differences in the rodent strain used; in the pattern, dose, and route of alcohol administration; and in the brain regions studied. In addition, the long-term behavioral changes that follow chronic intermittent alcohol exposure during adolescence may involve subtle changes in neuronal connections which are not easily measurable. Thus, additional research is necessary to elucidate the exact effects of alcohol on the adolescent hippocampus and other brain structures and to better understand the long-term implications of adolescent alcohol exposure.

## Footnotes

1 Human infants are born with far more neurons than are found in the adult brain. Based on a child’s interactions with the environment, the neurons and connections that are most meaningful can be selected.

2 Although there have been some demonstrations of “LTP-like phenomena” in the brain during certain types of learning, the term “LTP” itself refers to an experimentally induced change in brain function.

3 In normal brain function, nerve signals during memory formation are passed from other areas of the cortex to a region known as dentate gyrus, then to CA3 cells, CA1 cells, and finally back to the cortex.

4 Greater sensitivity of the glutamate/NMDA system in adolescents is not limited to the hippocampus but also is found in other regions of the cortex.
